# Does Cannabidiol Have a Benefit as a Supportive Care Drug in Cancer?

**DOI:** 10.1007/s11864-021-00934-0

**Published:** 2022-03-22

**Authors:** Sarah Lord, Janet Hardy, Phillip Good

**Affiliations:** 1 Mater Adult Hospital, Raymond Terrace, South Brisbane, QLD 4101 Australia; 2 Mater Research-University of Queensland, Brisbane, QLD Australia; 3 St Vincent’s Hospital, Brisbane, QLD Australia

**Keywords:** Cannabidiol, Supportive care, Advanced cancer, Cannabis

## Abstract

Cannabinoids have been purported as having a wide range of therapeutic uses although currently, there is minimal evidence to support these claims. Patients with advanced cancer experience many distressing symptoms, with some turning to medicinal cannabis to help alleviate these. Focus has fallen on cannabidiol (CBD) as a potential treatment for a variety of symptoms in advanced cancer due to the lack of psychoactive side effects and the potential molecular mechanisms of action associated with this cannabinoid. Many cannabinoid products are easily available in the community, and more countries are legalizing or allowing over the counter products. Studies show that CBD is generally well tolerated, but there are many potential drug interactions that have not been well studied. Few studies have specifically looked at the role of CBD in treating cancer symptoms, with most focusing on combination cannabinoid products. There are currently many unknowns associated with CBD, including which symptoms it might be best for, appropriate dosing, and route of administration. This is especially important in advanced cancer where patients often have significant organ dysfunction and frailty that could impact on the pharmacology of CBD. A small pilot study has shown promise for a role of CBD in the psychological symptoms associated with advanced cancer. Further research is currently underway to further clarify the role of CBD in this setting and to understand how best it might help our patients. Currently we advocate that CBD be used in supervised clinical trials, so that efficacy and adverse effects can be closely monitored.

## Introduction

Medicinal cannabis (MC) has gained significant attention in treating a wide variety of conditions, including symptoms associated with advanced cancer. Cancer patients experience a myriad of symptoms, many of which can be distressing. Not all respond well to conventional therapies [1]. The role of palliative and supportive therapy is to utilize a multidisciplinary approach to alleviate symptoms and improve quality of life. Many patients have turned to cannabis, medicinal, or otherwise, to help these symptoms, due to multiple anecdotal claims of benefit [2]. Public pressure has led to the legalization of medicinal cannabis in several countries despite the paucity of high-quality evidence for MC use in the palliative and supportive care setting.

Of all the known cannabinoids, cannabidiol (CBD) has gained particular interest as a potential symptom control drug due to its purported wide range of therapeutic targets. It has less psychoactive effects than other cannabinoids and thus is thought to be safer and more tolerable. It has, however, not been studied as extensively as other cannabinoids in clinical trials; therefore, its role is still yet to be fully understood. Much of the data used to back up its supportive role in cancer has been extrapolated from clinical trials of combination MC products or trials of CBD in other conditions.

## Endocannabinoids and cannabidiol pharmacology

Cannabis plants contain hundreds of bioactive compounds, with over 100 different cannabinoids. Delta-9-tetrahydrocannabionl (THC) and cannabidiol (CBD) have been the most extensively studied of these compounds. These plant-derived compounds interact with the endogenous cannabinoid signaling system in humans, the endocannabinoid system. The endocannabinoid system has only recently been characterized, as being strongly implicated in the nervous and immune system, with neuromodulatory, anti-inflammatory, and immunomodulatory properties [3]. The system regulates mood, appetite, memory, and sensation. The endogenous cannabinoids (endocannabinoids), such as anandamide and 2-arachidonoyl glycerol, are derived from arachidonic acid and are synthesized when the cannabinoid receptors are stimulated. Two major cannabinoid receptors are known, cannabinoid receptor type 1 and 2 (CB1 and CB2) [4]. They are G-protein-coupled receptors heavily expressed in the central and peripheral nervous systems. CB2 is also expressed in the spleen, hematopoietic cells, and immune tissues [5]. Activation of these receptors leads to a G-protein-coupled inhibition of excitatory neurotransmitter release in the brain and other signaling cascades in different tissues [3].

THC is the main psychoactive component of cannabis and is the best characterized. It binds to both CB1 and CB2 as a partial agonist, producing a wide variety of biochemical effects. It has been postulated to provide beneficial analgesic, antiemetic, and appetite stimulating effects. The many psychoactive effects of THC, including anxiety, sedation, cognitive impairment, hallucinations, and driving impairment, limit its use.

CBD was first structurally characterized in 1963, after initially being thought of as an inactive component of cannabis [3]. Unlike THC, CBD is not psychoactive and is now thought to have a wide range of therapeutic effects including antipsychotic, anxiolytic, anticonvulsant, anti-inflammatory, and analgesic. CBD has a low affinity for cannabinoid receptors, and any effects on these receptors appear to be indirect [6]. It is thought to act as a negative allosteric modulator at CB1 and CB2, reducing their response to other agonists, such as THC. It is through this mechanism that CBD is thought to reduce some of the psychoactive effects of THC, although a recent review of this in humans found inconsistent results [7]. CBD has been shown to interact with a variety of other receptors such as serotonin (5HT_1A_) receptors, transient receptor potential vanilloid (TRPV) channels, G-protein-coupled receptor 55, and peroxisome proliferator-activated receptor gamma (PPARγ) receptors [8]. The interaction of CBD with TRPV receptors is believed to lead to the anticonvulsant, antipsychotic, and immunomodulatory effects, whilst 5HT_1A_ actions mediate the anxiolytic and behavioral effects [9•]. CBD is metabolized in the liver by cytochrome P450 isoenzymes, primarily CYP2C19 and CYP3A4, and excreted in both urine and feces.

CBD can be administered orally, transcutaneously, sublingually, or vaporized. Most clinical studies have been carried out with the purified oral product Epidiolex™. Different sources of CBD, varying potencies, and different suspension agents are used in other studies making it difficult to compare and extrapolate the data. There are also significant gaps in the pharmacokinetic data of CBD in humans. Oral bioavailability has not been well studied in humans [10], but in animal studies, it is low. The time to maximum concentration (*T*_max_) after oral administration is variable, ranging from 1 to 6.12 h [11] with sublingual preparations having an earlier *T*_max_ than oral administration. This route of delivery would thus be expected to provide faster onset of action. The area under the curve and maximum concentration (*C*_max_) of CBD are dose dependent; therefore, increasing doses should produce greater effects [10,11]. *C*_max_, however, does not display a dose-dependent relationship in polydrug users [12], but the specific effect of opioids and other common palliative drugs on this has not been studied. Steady state is reached in roughly 2 days after starting administration [11]. The presence of food can increase CBD exposure compared to fasting in normal volunteers [11,13], which is of particular relevance in advanced cancer with many patients having poor oral intake. Hepatic impairment also increases the exposure to CBD [14], so patients with liver metastases may require lower doses to achieve similar therapeutic effects. As yet there are no studies that elucidate effective plasma concentrations of CBD, but there is a trend towards higher doses having better therapeutic outcomes [15]. Because of these incomplete data, there is no standard dosing of CBD, with the effective and tolerable dose range of CBD varying across studies from 20 to 6000mg a day. In a recent pilot study in advanced cancer patients, CBD doses were tolerated up to 600mg a day, with a median of 300mg/day [16••]. Common practice is to start at a low dose, titrating upwards to a desired response using oral products due to the more standardized bioavailability [17•].

## Safety and adverse effects of cannabidiol

CBD is generally considered to be a safe medication with few side effects, making it a good potential supportive therapy in cancer. As yet, there is no long-term safety data for CBD, with most clinical trials only assessing up to 14 weeks of use [18]. A 2020 review of the clinical safety data of CBD found an association between abnormal liver function tests, somnolence, sedation, and pneumonia in childhood epilepsy studies and found that CBD may interact with anti-epileptic medications [19]. After exclusion of the childhood epilepsy studies, the only adverse event documented as associated with CBD was diarrhea [19]. CBD has been shown to lead to an increased likelihood of withdrawal from studies over placebo, but again most of this data comes from childhood epilepsy studies, and extrapolation to advanced cancer is difficult. A recent pilot study in CBD in cancer patients receiving palliative care found the medication was generally well tolerated with the major adverse event being dose-related drowsiness, which improved with dose reduction [16••]. One of the major benefits of CBD over THC containing medications is that it does not impair driving ability [20] and, therefore, does not impact on the independence of patients. The risk of abuse is also thought to be low, with the World Health Organization’s (WHO) report into CBD finding no evidence of abuse potential [21].

Due to the liver metabolism by cytochrome P450 (CYP) isoenzymes, there is potential for many drug-drug interactions with CBD. It is well characterized in epilepsy that CBD interacts with common anti-epileptics, including clobazam, where it leads to an increase in serum levels [22] meaning that close observation is required. Concomitant CBD and sodium valproate use also affected liver function tests [22] necessitating increased monitoring. In cancer there are only minimal studies looking at the potential interactions of CBD with chemotherapy agents or other supportive medications. One study suggests that Cannabis tea usage does not interact with docetaxel or irinotecan, although both are CYP3A4 substrates and thus potentially affected by CBD. The immunomodulatory effects of CBD have led to some concern over interaction with many commonly used immunotherapy agents. An retrospective observation study looking at cancer patients treated with nivolumab showed a lower response rate in patients using several cannabis products containing CBD [23]. Case reports are also arising of significant drug-drug interactions of CBD, including one in a child on methadone resulting in a clinically significant increase in the serum methadone levels [24]. This highlights the need for more investigation into potential interactions of CBD with cancer and supportive treatments.

## Current use and availability

It is estimated that between 4 and 24% of advanced cancer patients are already using cannabis of some form [25,26,27,28]. One study in America reported that 24% of palliative patients surveyed, 67% with cancer, were using CBD. All had started using CBD due to their illness [28]. Most CBD use is not prescribed but sourced through other means, often illegally or in permitted over the counter products [27]. As most studies rely on either self-reporting or urine drug testing for THC, it is likely that the number using CBD is actually higher, especially now that CBD-containing products are more easily available. Studies have shown a variety of reasons for cannabis use in cancer, but the management of pain, nausea, and anxiety feature heavily [25,29]. Patients with cancer who use cannabis have also been shown to have a higher symptom burden than non-users [27], which could indicate that people turn to cannabis due to failure of other treatments. Despite no evidence that cannabis can cure or even slow the progression of cancer in human studies, patients often believe that cannabis provides them with a hope of cure [28, 29]. This is backed up by strong media publication of stories claiming miraculous effects [2].

MC is available in a variety of preparations, which contains either pure THC or a combination of THC and CBD (B2). Pure CBD products are now available with the approval of Epidiolex™, a 100mg/mL CBD oil, by the Food Drug Administration (FDA) in America and the European Medicines Agency (EMA). Sativex™, which is a buccal nabiximols spray containing a 1:1 combination of THC:CBD, is also a registered product available on prescription in many countries, delivering low-dose CBD. In 2019 the WHO stated that CBD preparations should not be subject to international drug control, as CBD is not intoxicating, well tolerated, and associated with low abuse potential [21]. This has made way for more countries to relax controls on CBD products, and now many are available over the counter, although the products are often very low dose [30]. There is much concern about the content of these products [31] as they are often inaccurately labeled [32]. They may contain a variable amount of the CBD, as well as other cannabinoids, especially THC, in higher quantities than the label suggests. This is particularly troublesome in countries where it is illegal to drive after consuming THC, and patients need to be counseled on this.

## CBD and cancer-related symptoms

Most of the current clinical evidence for CBD use is focused on epilepsy and childhood seizure disorders, with small numbers of trials focused on other areas including chronic pain. Most trials of medicinal cannabis in cancer and cancer-related symptoms involve THC or combination products. There is currently no randomized controlled trial (RCT) published in the palliative setting assessing the effect of CBD on symptoms; however, several trials are currently underway [33,34]. In this section, we discuss the available evidence surrounding several of the more common symptoms of advanced cancer and the possible role of CBD.

### CBD and pain

Pain is one of the most studied symptoms in cannabis reports, with strong anecdotal evidence of benefit that has not been replicated in high-quality clinical trials. Only a few studies have looked at the role of CBD specifically, and there have been no RCTs of pure CBD products in pain management in cancer. Several potential molecular mechanisms for the analgesic effect of CBD have been studied in mouse models, including in neuropathic and taxol-induced pain, with promising analgesic affects seen [35]. However, this has not yet been replicated in human studies.

Several early studies assessed CBD in pain primarily due to multiple sclerosis (MS) or nerve injuries. A randomized, double-blind, placebo-controlled, crossover study, using CBD sublingual spray (2.5–120mg/day) in 20 patients with MS, spinal injury, brachial plexus lesions, or amputation, showed a decrease in pain measured on a visual analog scale [36]. In contrast a 2.5 mg sublingual CBD spray for 8 weeks did not improve chronic pain on a visual analog score in patients with chronic pain primarily due to MS [37]. CBD has also been shown to lack analgesic effect in fibromyalgia [38]. These studies are limited by the small size and low dose of CBD used.

More recently a randomized, double-blind, crossover study looking at topical CBD, 250mg/dose up to 4 times a day, in neuropathic pain reported a larger decrease in ratings of “intense, sharp, cold, and itchy” on the Neuropathic Pain Scale in the CBD groups compared to placebo [39]. This suggests that there may be a role for CBD in neuropathic pain, but again, the study was small and included a variety of etiologies, making it hard to know how transferable this is to advanced cancer-related neuropathic pain. A small cross-sectional study looking at THC and CBD use in the outpatient palliative setting found that 50% of patients taking CBD reported improvements in their pain [28]. A pilot study by Good et al. looking at CBD and THC in advanced cancer found no difference in pain score from baseline at day 14 [16••]. This study was small but has led to a larger placebo controlled of CBD in this setting being undertaken [33].

No convincing data has been produced in humans that CBD can be opioid sparing. In a single-arm study looking at chronic pain patients, of indeterminate cause, with moderate-high-dose opioid use (>50mg day oral morphine equivalent), treated with CBD-rich topical hemp extract, 50 patients self-reported a reduction in opioid medications at 8 weeks [40] suggesting an analgesic effect. This reduction was not able to be quantified and included those deliberately missing doses rather than a prescribed decrease in opioids; therefore, this data must be interpreted with caution. In a current RCT of CBD versus placebo in over 100 cancer patients, a few participants have reduced their use of breakthrough medications, but no participant to date has reduced their baseline opioid dose [33].

Combination products of CBD and THC have been more extensively studied in pain, with several studies in advanced cancer. Two multinational studies of nabiximols in cancer have shown no superiority over placebo in reducing self-reported pain scores [41,42]. A recent systematic review and meta-analysis of 5 RCTs looking at cannabinoids in cancer pain found no change in average numerical pain scores between cannabinoids and placebo, with a higher risk of adverse events with cannabinoids [43]. Another systematic review of cannabis and cannabis medicines for pain of all types found that although there is a trend towards efficacy, the studies are of low quality, and therefore they concluded they could not support or refute any claims of benefit [44,45]. Again this highlights the need for further, high-quality data in this area. Although the NASEM claims benefit in pain control [46], especially in neuropathic pain, cancer guidelines emphasize that MC should never be used first line in the management of cancer pain [47]. At present there is no data to support using CBD or combination products as a first-line treatment for cancer pain, and its role as an adjunct, especially in neuropathic pain, needs further clarification and study.

### CBD and nausea

Nausea is a common symptom in advanced cancer. It is often associated with cancer treatment or to the disease itself. CBD appears to be an attractive candidate to manage chemotherapy-induced nausea due to its modulation of the 5-HT_3_ receptors. Chemotherapy induces increased serotonin release, activating 5-HT_3_ receptors on vagal nerve afferents that mediate emesis signals in the brain. Whilst there is anecdotal evidence for CBD as an antiemetic, there are no studies looking specifically at CBD alone and its effect on nausea of any type.

Combination therapies (THC:CBD) have shown some promise against placebo in chemotherapy-induced nausea in older studies, but few studies have looked at cannabis products compared to modern anti-emetics. A recent phase 2 crossover study has compared 1:1 THC:CBD capsules to placebo in addition to standard anti-emetics in resistant nausea due to chemotherapy. Oral THC:CBD led to less nausea but more side effects [48], and a larger phase 3 study is currently underway. Interesting several of the RCTs in pain reported greater nausea and vomiting in the THC:CBD group as an adverse event or secondary outcome [41, 42, 49, 50]. No difference in nausea was found in the Good et al. pilot study of CBD or THC alone in advanced cancer at 14 days after initiation [16••]. There are currently no studies assessing cannabis products for nausea in advanced cancer that is not directly due to chemotherapy or on the role of CBD alone in this cohort.

### CBD and appetite

Decreased appetite and anorexia are amongst the most common and distressing symptoms of advanced cancer [1]. At present there are only a limited number of medications that have any evidence for benefit in this area. Cannabis has been considered a possible treatment, as endocannabinoids are known to be involved in eating behavior. Benefit of medicinal cannabis on appetite has been seen in HIV [51]. However, studies of THC and cannabis extract in cancer have failed to show any benefit over placebo in increasing appetite [52,53]. Studies examining combination products for pain have reported negative effects on appetite as a secondary outcome measure [41]. A small survey of CBD use in palliative patients found that 29% self-reported an increase in appetite [28]. The pilot study by Good et al. also found no significant effect on appetite with CBD or THC in cancer. No randomized studies have primarily looked at CBD and its effect on appetite or weight gain in cancer

A recent review analyzed the effect of oral CBD on decreased appetite reported as an adverse event in trials for epilepsy and schizophrenia [54]. Doses of CBD ranged from 20 to 1000 mg/day. In 4 of the 6 RCTs evaluated, CBD was shown to decrease appetite, with no effect on appetite in the other 2 studies. Although this patient cohort is very different to those with advanced cancer, it would appear that CBD might actually be detrimental to appetite in our patients rather than beneficial. Further studies powered to investigate this are required.

### CBD and sleep

Endocannabinoids are involved in regulation of the circadian rhythms in humans, especially in the maintenance and promotion of sleep; therefore, cannabis products provide a potential therapy to aid the debilitating symptoms of poor sleep and associated fatigue in advanced cancer patients. They are also purported to improve sleep through anxiolytic effects. CBD has a safety advantage over classical hypnotic medications (that often interact with other supportive medications), therefore, making it a promising candidate. However, the acute and chronic effects of CBD on sleep are not clearly understood.

Observational studies report patients feel CBD is effective for improving sleep [55], yet the RCT data is lacking. Despite promising pre-clinical evidence, a recent systematic review of cannabinoids for sleep disorders found there is currently insufficient evidence to support any cannabis products for sleep [56]. Several other reviews looking at cannabis and combination products effects on sleep source their data from studies powered to assess the effect of cannabis on pain [49, 50], with variable effects on sleep as a secondary outcome [57, 58]. Looking at sleep as an adverse event in CBD studies for epilepsy, schizophrenia, drug dependence, and chronic pain, no reduction in sleep quality was found [54] suggesting that in these populations, CBD has at least a neutral effect on sleep. A study in chronic pain, looking at CBD-rich hemp extract, found that sleep quality significantly improved at 4 and 8 weeks as a secondary outcome [40]. Again, a small survey of CBD use in palliative care patients found a 29% self-reported improvement in insomnia after taking CBD [28]. There is no RCT examining the effects of cannabis or CBD on sleep in cancer.

### CBD and psychological symptoms

Anxiety and stress relief are common reasons for CBD to be used in the general population [52], with similar patterns seen in the cancer population. Pre-clinical evidence suggests that CBD may have an anxiolytic effect and that it counteracts the psychoactive effects of THC. The role of CBD as a clinical anxiolytic has been studied in a variety of settings including generalized anxiety disorder, social phobia, schizophrenia, and healthy adults. No studies have primarily assessed the role of cannabis or CBD in anxiety or depression in cancer patients. There is moderate evidence that single-dose CBD can reduce anxiety after a simulated public speaking test in healthy volunteers and those with social phobia [9•]. There is also some evidence that it reduces anxiety in generalized anxiety disorders. Overall, the clinical evidence is mixed, but the studies are hard to interpret due to the differing dose ranges and regimens of CBD used [9•].

A recent open label pilot study looking at cannabinoids in palliative care found a significant improvement on the emotional subscale of the ESAS (Edmonton symptom assessment scale), with anxiety and depression being the individual symptoms with the greatest improvement [16••], with 16 patients out of 21 taking CBD. The same pilot study reported a decrease in the median DASS 21 (depression, anxiety, and stress scale) scores, with the depression and stress sub-scores significantly decreasing. A subsequent randomized controlled trial looking at CBD in palliative patients is underway to test the true benefit in this population.

### CBD and quality of life

Improvement in overall quality of life is the main goal of supportive care in advanced cancer, and theoretically, CBD shows a great promise to do so. However, few studies have evaluated the effect of medicinal cannabis on overall quality of life. A systemic review of medicinal cannabis products found no significant effect on health-related quality of life when all medical conditions were included [59]. Good et al. found that 44% of advanced cancer patients reported an overall improvement in their condition since starting cannabis when measured using the PGIC (patient global impression of change) scores, but there was no changes in overall quality of life as measured by the EORTC quality of life measure [16••]. Interestingly, a cohort study looking at CBD-rich hemp in chronic pain found that 94% of patients reported improved quality of life on subjective, open-ended questions, but formal quality of life indices or questionnaires showed no significant changes [40]. No studies to date have looked at the effect of CBD alone on quality of life in cancer.

## Limitations and the future

CBD is an attractive candidate in the palliative setting as it is well tolerated with potentially many beneficial biochemical effects. As outlined above, however, there is a paucity of evidence of its benefit in managing the symptoms associated with advanced cancer. One of the difficulties in interpretation of the evidence is the wide variety of formulations, doses, and dosing intervals used in studies and the heterogeneous populations under study. This further complicates the ability of clinicians to prescribe CBD and explains the common physician hesitation around its use. Monitoring longer term use is especially important in populations such as those with cancer, where patients have a multitude of comorbidities and biochemical abnormalities, especially as potential toxicities and drug reactions are possible. Future research should focus on which symptoms, if any, are best palliated by CBD, at what dose, and by which route, so that a more standardized approach can be taken. This would also enable physicians to feel more comfortable in prescribing these products and allow patients to be better educated about its role in their care.
